# Indoor Smartphone Localization Based on LOS and NLOS Identification

**DOI:** 10.3390/s18113987

**Published:** 2018-11-16

**Authors:** Hyeon Jeong Jo, Seungku Kim

**Affiliations:** School of Electronics Engineering, Chungbuk National University, Chungbuk 28644, Korea; johj0840@gmail.com

**Keywords:** indoor localization, LOS/NLOS, smartphone, trilateration, WiFi

## Abstract

Accurate localization technology is essential for providing location-based services. Global positioning system (GPS) is a typical localization technology that has been used in various fields. However, various indoor localization techniques are required because GPS signals cannot be received in indoor environments. Typical indoor localization methods use the time of arrival, angle of arrival, or the strength of the wireless communication signal to determine the location. In this paper, we propose an indoor localization scheme using signal strength that can be easily implemented in a smartphone. The proposed algorithm uses a trilateration method to estimate the position of the smartphone. The accuracy of the trilateration method depends on the distance estimation error. We first determine whether the propagation path is line-of-sight (LOS) or non-line-of-sight (NLOS), and distance estimation is performed accordingly. This LOS and NLOS identification method decreases the distance estimation error. The proposed algorithm is implemented as a smartphone application. The experimental results show that distance estimation error is significantly reduced, resulting in accurate localization.

## 1. Introduction

The number of smart devices has increased exponentially with the development of information and communication technology. The number of worldwide smartphone users reached 3.9 billion in November 2016, which is more than 50% of the world’s population. The smartphone combines various communication technologies and sensors that enable the provision of location-based services (LBS) [1]. LBS applications were mainly developed in the military and naval areas, but the smartphone has recently served many private LBS applications. Typical applications are indoor navigation, the control of unmanned robots, augmented reality games, logistics tracking systems, and location-based advertisements (LBA). In the future, the LBS is expected to be used in a broader set of fields.

Accurate localization technology is essential for the provision of robust LBS. GPS is a typical localization technique using satellite signals [2]. Most smartphones are equipped with a GPS module. Hence, smartphone users can easily obtain their location information. However, GPS cannot offer indoor location information because satellite signals cannot penetrate walls. For this reason, LBS applications requiring indoor location information cannot use GPS-based localization. Various studies have been done on the accurate recognition of indoor position. Pedestrian dead reckoning (PDR) is a typical indoor localization technology that uses inertial measurement units (IMU), such as accelerometers, gyroscopes, and magnetometers. The PDR uses IMU sensors to infer the user’s position by estimating the user’s step count, stride, and heading direction [3]. Many PDR approaches estimate a user’s location based on a previously inferred location. These approaches generate large errors due to the accumulation of many small errors of the sensors involved in the localization algorithms. Therefore, the combination of location calibration techniques with other technologies are necessary for PDR. Geomagnetism-based indoor localization is a technique used to estimate a user’s position using a magnetic field map at each location [4]. In the indoor environment, each location has its own magnetic field characteristics because the building materials and structures distort the Earth’s magnetic field. By means of these characteristics, a magnetic field map is first constructed for each position. The user’s location can be estimated from a corresponding pre-configured magnetic field map. This method requires time and money to construct a magnetic field map in advance, and moving objects, such as cars or elevators, transform this map. For this reason, geomagnetism-based indoor localization is difficult to apply practice.

Wireless communication technologies, such as WiFi, ZigBee, and Bluetooth, can be used for indoor localization. The received signal strength indicator (RSSI) indicates the power received from the radio signal by a receiver. A fingerprint technique uses the distribution pattern of the RSSIs [5]. It divides the area into several cells and constructs an RSSI map stored in a dedicated database. The user’s location is then obtained by comparing the RSSI map with the user’s RSSI. As the size of the cell becomes small, the fingerprint technique provides accurate localization. However, the construction of an RSSI map in advance requires significant time and money. In addition, RSSI maps are unreliable, because RSSIs are very sensitive to environmental changes, including temperature, humidity, and obstacles. Another localization method using wireless communication is the estimation of the distance or direction between a sender and a receiver using the characteristics of radio signals. The path loss model [6] allows the estimation of a relative distance using RSSIs. Time of arrival (TOA) uses the propagation time of a radio signal from a sender to a receiver. It is possible to estimate the distance through TOA using a known radio propagation speed [6]. TOA-based localization requires accurate time synchronization between a sender and a receiver. Angle of arrival (AOA) measurements determine the direction of a radio signal using an array of antennas [6]. We can generally choose WiFi or Bluetooth as the candidate wireless communication technology for smartphone-based indoor localization. Recent smartphones are not equipped with an array antenna for wireless communication. AoA localization is applicable by means of an access point (AP) equipped with an array antenna or multiple antennas, but APs should include the AoA localization algorithm. In other words, it is difficult to implement the AoA localization with a smartphone application alone. Moreover, the AoA algorithm requires the line-of-sight (LOS) propagation path. The RSSI-based and TOA-based distance measurements require a line-of-sight path from the smartphone to WiFi access points (APs) or Bluetooth beacons in order to yield low distance errors. We study RSSI-based localization, which is simpler to implement than TOA-based localization.

WiFi is a wireless technology for local area networking based on IEEE 802.11 standards. WiFi APs are accessible nearly everywhere. The latest smartphones are equipped with a WiFi module that operates in the 2.4 and 5 GHz bands. By leveraging WiFi technology that is already deployed, we can recognize indoor locations without the extra costs required for building infrastructure.

In this study, we propose an indoor smartphone localization algorithm based on accurate distance measurements using WiFi RSSIs. The propagation paths of radio signals can be line-of-sight (LOS) or non-line-of-sight (NLOS) in an indoor environment. In the LOS path, RSSIs are mainly affected by distance, so accurate distance estimation is possible. On the other hand, in the NLOS path, the radio signals are attenuated not only by distance, but also by obstacles, including walls, columns, furniture, and humans. This signal attenuation causes a distance estimation error when using the conventional distance estimation model. If we do not consider the characteristics of the propagation path, the estimated distance error increases, resulting in inaccurate localization. To overcome this problem, the proposed localization algorithm first classifies the propagation path as LOS and NLOS using the attenuation characteristics of the 2.4 GHz and 5 GHz bands [7]. Based on this distance estimation, we implement a trilateration mechanism to obtain the position of the smartphone. Our main contributions are summarized as follows:

- We present a novel indoor localization algorithm that does not require extra hardware or infrastructure. It is a practical technology that can be applied to any hardware platform.
- We define a theoretical method to distinguish between LOS and NLOS. This increases the level of accuracy in the estimated distance, so it is possible to reduce the localization error in the trilateration mechanism.
- We implement the proposed localization algorithm as a real Android application. In the experiments, the localization error shows that, in practice, it has a viable performance for distances of less than 1.5 m.

The rest of this paper is organized as follows. Section 2 introduces background information on localization. Section 3 demonstrates the proposed indoor localization algorithm. Section 4 introduces some related research. Section 5 evaluates the performance of the proposed localization algorithm, and Section 6 presents our conclusions.

## 2. Background

### 2.1. Propagation Model

The RSSI is the measured power of a propagated radio signal at the receiver. RSSIs are influenced by small-scale and large-scale fading [8]. Small-scale fading includes multipath fading effects that reflect, diffract, and scatter the propagated signals by obstacles, as well as the Doppler effect that changes the frequency of the propagated signals due to motion of the object. Large-scale fading contains path loss effects according to the distance and shadowing due to reflection and diffraction by obstacles. For indoor localization, a lower number of errors in distance estimation occurs in small-scale fading than in large-scale fading [9]. Therefore, this paper does not consider the effect of small-scale fading.

The *Friis* transmission equation gives the power received by the receiver’s antenna under ideal conditions [10]. The *Friis* transmission equation is as follows:

(1) $$\;P_{Rx}=\frac{P_{Tx}G_{Tx}G_{Tx}\lambda^{2}}{{\left(4\pi d\right)}^{2}}\;$$

where *P_Rx_* and *P_Tx_* are received and transmitted power, respectively. *G_Tx_* and *G_Rx_* indicate the antenna gains of the sender and the receiver, respectively. *λ* is the wavelength of the radio signal, and *d* is the distance between the sender and the receiver. As shown in Equation (1), the received power is proportional to *λ*^2^ and inversely proportional to *d*. In other words, an RSSI decreases as the propagation distance becomes longer and the communication frequency band increases. The wavelengths in the 2.4 GHz and 5 GHz band are 12.5 cm and 6 cm, respectively, thus the RSSI at 2.4 GHz is larger than that at 5 GHz when the transmitted power and propagation distance are identical. For ideal isotropic antennas, the Equation (1) can be simplified to

(2) $$\frac{P_{Rx}}{P_{Tx}}={\left(\frac{\lambda }{4\pi d}\right)}^{2}.$$

If the propagation path is free space with a 1 m reference distance, Equation (2) can be expressed as the path loss in terms of *dB*:

(3) $$\;PL_{free\;space}=20\log_{10}\left(f\right)+20\log_{10}\left(d\right)+32.44\;$$

where *f* and *d* are the frequency in MHz and the propagation distance in meters, respectively. From Equation (3), it can be observed that the path loss is determined by the frequency. The *Friis* transmission equation is a radio propagation model that is used in free space. The indoor environment is not free space; hence, another path loss model should be used for indoor localization.

The log-distance path loss model is a modification of the *Friis* transmission equation, where additional factors are included based on the particular indoor environment [11]:

(4) $$\;PL=P_{Tx_{dB}}-P_{Rx_{dB}}=20\log_{10}\left(\frac{4\pi d_{0}}{\lambda }\right)+10n\log_{10}\left(\frac{d}{d_{0}}\right)=PL_{0}+10n\log_{10}\left(\frac{d}{d_{0}}\right)\;$$

where *PL* is the path loss measured in *dB*. *PL_0_* is the path loss at a reference distance *d_0_*, and *n* is the path loss exponent. The propagation distance *d* can be derived from Equation (4). The path loss exponent is a function of the carrier frequency, propagation environment, obstacles, etc. It generally varies from 1.2 to 6, i.e., 1.8 for indoor, 2 for free space, and 3 to 5 in an urban area [12]. In the indoor environment, the internal structure acts as a waveguide; hence, it is less than the propagation path for free space, i.e., 2. The path loss at the reference distance *PL*_0_ can be replaced with $P_{Tx0_{dB}}-P_{Rx0_{dB}}$ in Equation (4), and we can derive Equation (5) in terms of the received power:

(5) $$P_{Rx_{dB}}=P_{Tx_{dB}}-P_{Tx0_{dB}}+P_{Rx0_{dB}}-10n\log_{10}\left(\frac{d}{d_{0}}\right).$$

$P_{Tx_{dB}}$ and $P_{Tx0_{dB}}$ are the transmitted powers, which do not depend on distance. Because they have the same value, Equation (5) can be simplified as follows:

(6) $$P_{Rx_{dB}}=P_{Rx0_{dB}}-10n\log_{10}\left(\frac{d}{d_{0}}\right).$$

If we assume the reference distance *d_0_* is 1 m, then Equation (6) can be expressed in terms of *d*:

(7) $$d=10^{\frac{P_{Rx0_{dB}}-P_{Rx_{dB}}}{10n}}.$$

In this study, we used Equation (7) to estimate the distance.

### 2.2. Trilateration Localization

**Figure 1:**
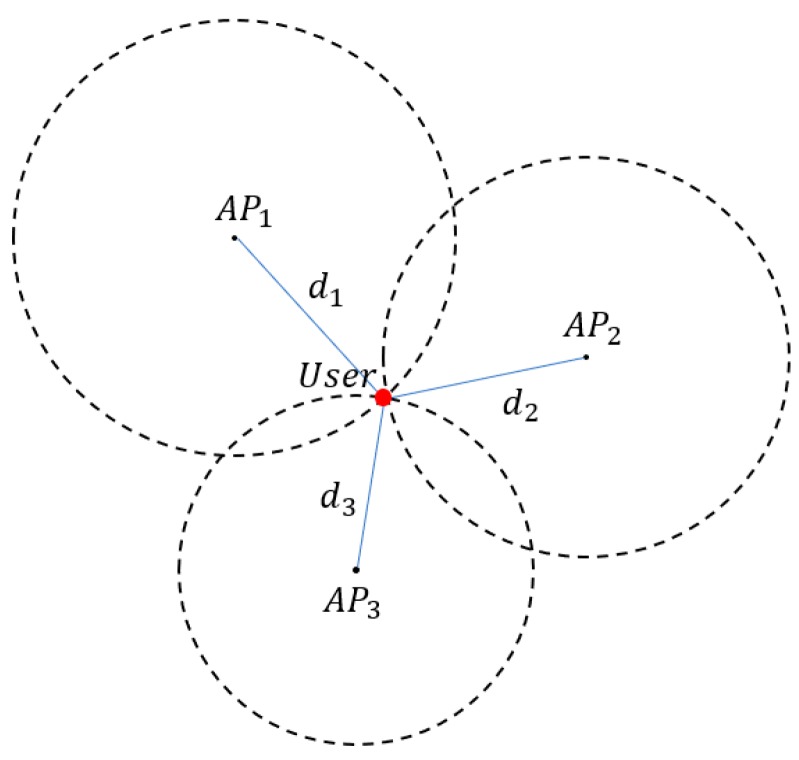
A sample of trilateration localization.

Trilateration is the most typical method for localization. It uses the distance among devices in a two-dimensional plane, as shown in Figure 1. The coordinates of *AP_n_* and a user are denoted as (*x_n_*, *y_n_*) and (*x*, *y*), respectively. The distance between them is *d_n_*, and this can be derived from Equation (7).

The distance to each AP can be expressed as follows:

(8) $$\;d_{1}{}^{2}={\left(x-x_{1}\right)}^{2}+{\left(y-y_{1}\right)}^{2}\;\;d_{2}{}^{2}={\left(x-x_{2}\right)}^{2}+{\left(y-y_{2}\right)}^{2}\;d_{3}{}^{2}={\left(x-x_{3}\right)}^{2}+{\left(y-y_{3}\right)}^{2}.$$

From Equation (8), we can obtain a linear equation connecting the intersection of two circles as follows:

(9) $$2\left(x_{1}-x_{2}\right)x+2\left(y_{1}-y_{2}\right)=x_{1}{}^{2}-x_{2}{}^{2}+y_{1}{}^{2}-y_{2}{}^{2}-d_{1}{}^{2}+d_{2}{}^{2}\;2\left(x_{2}-x_{3}\right)x+2\left(y_{2}-y_{3}\right)=x_{2}{}^{2}-x_{3}{}^{2}+y_{2}{}^{2}-y_{3}{}^{2}-d_{2}{}^{2}+d_{3}{}^{2}.$$

The above equation can be expressed as a matrix:

(10) $$A_{2,2}X=B_{2,1},\;\mathrm{where}\;A_{2,2}=\left[\begin{array}{cc} 2\left(x_{1}-x_{2}\right) & 2\left(y_{1}-y_{2}\right)\\ 2\left(x_{2}-x_{3}\right) & 2\left(y_{2}-y_{3}\right) \end{array}\right]{,}^{}X=\left[\begin{array}{c} x\\ y \end{array}\right],\;B_{2,1}=\left[\begin{array}{c} x_{1}{}^{2}-x_{2}{}^{2}+y_{1}{}^{2}-y_{2}{}^{2}-d_{1}{}^{2}+d_{2}{}^{2}\\ x_{2}{}^{2}-x_{3}{}^{2}+y_{2}{}^{2}-y_{3}{}^{2}-d_{2}{}^{2}+d_{3}{}^{2} \end{array}\right].$$

By multiplying both sides by the inverse of *A*, Equation (10) can be transformed into:

(11) $$\;X=A_{2,2}{}^{-1}B_{2,1}\;$$

where *X* represents the estimated position of the user.

## 3. Proposed Indoor Localization Algorithm

The most challenging issue in RSSI-based localization is decreasing the estimated distance error. The propagation model is an applicable method when the radio signal propagates along a straight line. However, the radio signal can be propagated through multiple paths in an indoor environment, which causes large-scale fading [13]. For this reason, RSSIs do not always have a constant value over the same distance. Such a distance error causes inaccurate location estimation. The proposed localization algorithm first distinguishes RSSIs received under LOS from RSSIs received under NLOS. Most smartphones include WiFi technology capable of communicating in the 2.4 GHz and 5 GHz bands. Our algorithm uses the characteristics of each frequency band to distinguish between LOS and NLOS signals. Through the classification of LOS and NLOS, we can estimate a relatively accurate distance. The user’s location is determined using a trilateration method based on the estimated distance.

### 3.1. Preliminary

**Figure 2:**
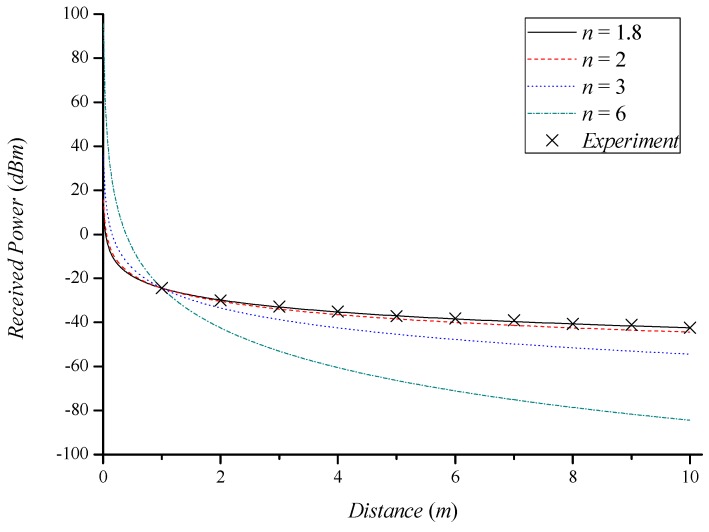
Received power as a function of distance with different path loss exponents.

Figure 2 shows the RSSI as a function of distance, where each line is derived by Equation (6), and X marks indicate the experimental results in the LOS propagation path. The experimental environment was the same as for the performance evaluation in Section 5. As the path loss exponent becomes larger, the received power difference increases with distance. This proves the effect of shadowing on the received power. The experimental result in the LOS propagation path and the theoretical result where *n* is 1.8 are similar to each other. This verifies that the real received power follows the log-distance path loss model.

Path loss exponents are different and depend on the specific propagation path. In a LOS environment, the path loss is mainly affected by distance. The path loss in an NLOS environment is determined by the shadowing effect as well as distance; hence, the NLOS path loss exponent is larger than the LOS path loss exponent. Since the path loss exponent determines the distance in the path loss model, it is very important to choose a suitable path loss exponent for a particular propagation path.

**Figure 3:**
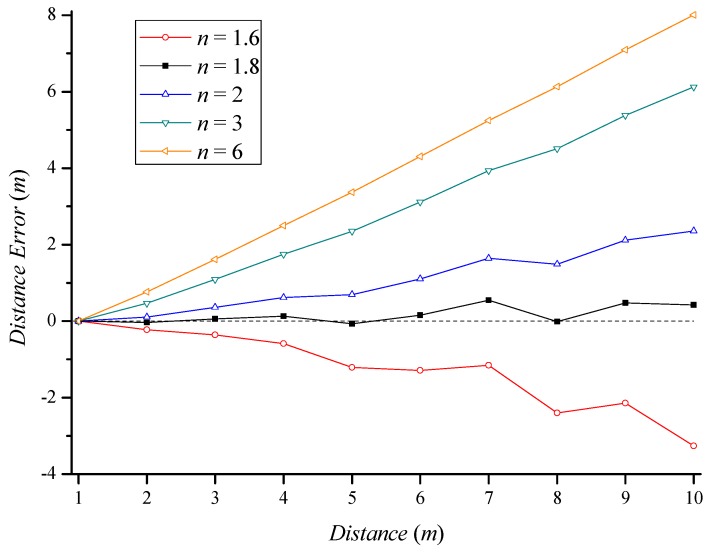
Estimated distance error in an indoor line-of-sight (LOS) environment according to path loss exponents.

Figure 3 shows the distance errors for different path loss exponents as a function of distance. We constructed an indoor LOS propagation path to collect RSSIs. The RSSI value collected at each distance was substituted into Equation (7) to obtain the estimated distance for different path loss exponents. As shown in Figure 2, the path loss exponent in an indoor environment was close to 1.8. As *n* used for the distance estimation was closer to the path loss exponent of the experimental environment, the distance estimation error was smaller. The experimental result shows that the smallest distance error occurred when we selected a path loss exponent of 1.8 for the distance estimation. This result verifies that selecting the path loss exponent corresponding to the propagation path is important for increasing the accuracy of distance estimation.

### 3.2. LOS and NLOS Identification Method

In order to distinguish between LOS or NLOS propagation paths, we exploited the differences in propagation characteristics between the 2.4 GHz and 5 GHz bands, as was used in [7]. As described in the propagation model in Section 2, the receiver obtains different RSSIs for the same output power depending on the wavelength of the radio signal. When 2.4 GHz and 5 GHz radio signals are transmitted from the same AP, the path loss difference △*PL* between the two signals can be derived from Equation (6):

(12) $$\begin{array}{cc} \Delta PL & =P_{Rx_{2.4GHz}}-P_{Rx_{5GHz}}\\ & =P_{Rx0_{2.4GHz}}-P_{Rx0_{5GHz}}-10\left(n_{2.4GHz}-n_{5GHz}\right)\log_{10}\left(\frac{d}{d_{0}}\right)\\ & =\Delta PL_{0}-10\left(n_{2.4GHz}-n_{5GHz}\right)\log_{10}\left(\frac{d}{d_{0}}\right)\; \end{array}$$

where $P_{Rx0_{2.4GHz}}$ and $P_{Rx0_{5GHz}}$ are the levels of received power in the 2.4 GHz and 5 GHz bands over the reference distance, respectively. The path loss difference due to signal attenuation of these two bands depends on the path loss exponent *n* for each band if their distances *d* are identical. Because the wavelength of a 5 GHz single is shorter than that for a 2.4 GHz signal, the 5 GHz signal generally experiences greater attenuation. As described in [14], the path loss exponents for the 2.4 GHz and 5 GHz bands are not significantly different in an indoor LOS environment. This means that the difference in signal attenuation due to distance in both bands is very low. In this paper, we determined LOS and NLOS using the path loss difference, △*PL*.

### 3.3. Indoor Localization Algorithm

**Figure 4:**
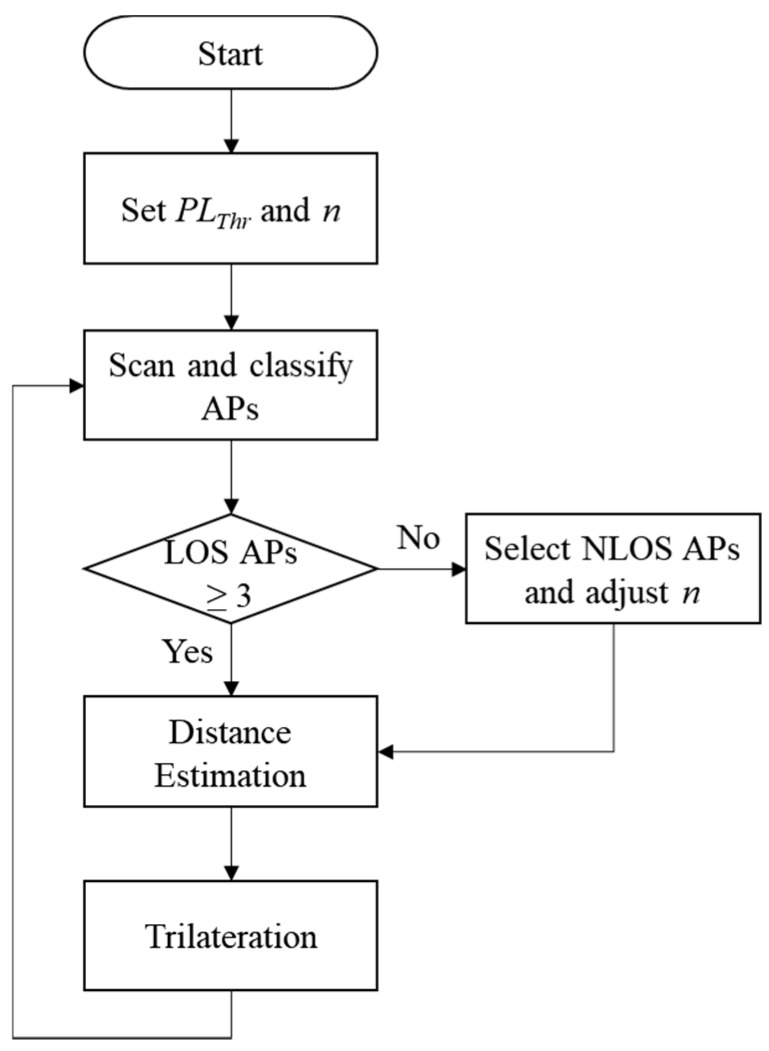
The proposed indoor localization algorithm.

In an indoor environment, many WiFi APs periodically transmit beacon frames to each channel in order to inform nearby WiFi stations of their presence (e.g., smartphones). We used WiFi technology for indoor localization, which does not require additional infrastructure. Figure 4 shows the flowchart of the proposed indoor localization algorithm. Before starting the indoor localization, the smartphone first sets path loss thresholds and path loss exponents according to each propagation path. According to the path loss difference in Equation (12), the smartphone determines the propagation path with each AP. The propagation path is classified as follows:

(13) $$\;\{\begin{array}{cc} \;\Delta PL\leq PL_{Thr1} & for\;LOS\\ PL_{Thr1}<\Delta PL\leq PL_{Thr2} & for\;NLOS\;Lv.\;1\\ PL_{Thr2}<\Delta PL\leq PL_{Thr3} & for\;NLOS\;Lv.\;2 \end{array}\;$$

where *PL_Thr_* is a predefined path loss threshold at the smartphone. Any APs whose path loss difference exceeds *PL_Thr3_* is not used for localization. This reduces the situation where the trilateration is not possible due to distance estimation error (i.e., no intersection among the APs). In this paper, NLOS level 1 was defined as the propagation path with one concrete wall and NLOS level 2 was defined as the propagation path with two concrete walls.

The smartphone scans APs by collecting beacons from APs and classifies them as either LOS or NLOS using Equation (13). The smartphone classifies and stores APs with path loss of *PL_Thr3_* or less for localization. In cases where three or more LOS APs are nearby, the smartphone immediately estimates the distance to the APs and uses trilateration to estimate its location. Three LOS APs allow accurate localization through low distance estimation error. Even if many WiFi APs exist around a smartphone, it is often impossible to find more than three LOS APs indoors. This is because the indoor environment is composed of various obstacles, like walls. If less than three LOS APs are nearby, level 1 and 2 NLOS APs participate in localization. In this case, the smartphone selects the NLOS APs in descending order of path loss difference. If no intersection among the radio circle of three APs exists, the trilateration cannot be a solution for the localization. In this case, the gravity of the triangle with three APs is determined as a line which is judged as the estimated location, but this cannot guarantee an acceptable level of error. To estimate the distance using the NLOS AP, the path loss exponent is adjusted according to the status of the propagation path. Appropriate path loss exponents are heuristically obtained from experiments. Even if the proper path loss exponent according to the propagation path is used, the accuracy of distance estimation is lower when NLOS paths are used. Therefore, the localization accuracy is determined by the number of LOS APs.

## 4. Related Works

Many studies have strived to improve the accuracy of RSSI-based indoor localization. RSSI-based indoor localization is classified into fingerprinting and trilateration [15]. Many works have proposed fingerprinting-based localization, because it is hard to apply a propagation model in trilateration-based localization due to the fading effect. Recently, a more sophisticated fingerprinting-based localization using channel state information (CSI) was proposed. The CSI is more reliable than the RSSIs that change frequently depending on the time and environment. The fingerprint map is constructed through CSI and deep learning is applied to the training step to improve the reliability of the localization [16,17,18]. However, trilateration-based localization has advantages in practical use because it does not require a costly background survey and database maintenance for radio map. The main purpose of this paper was to achieve smartphone localization by using the existing infrastructure. We introduced typical trilateration-based localization algorithms and LOS/NLOS identification methods to reduce the distance estimation error.

Zheng et al. [19] proposed a quality of trilateration (QoT) algorithm that reduces the error of trilateration in a noisy environment. Because RSSI is sensitive to noise, the estimated distance includes error, resulting in inaccurate localization. The QoT assigns a confidence value to each node and selects nodes with high confidence values, thereby reducing the localization error. However, this probabilistic approach requires a large number of nodes to be used for localization. Our main research objective was to enhance practicality, so we did not consider this probabilistic approach. Dag et al. [20] presented the RSSI-based least squares lateration algorithm, which uses more than three APs for indoor localization. They compared the performance when three APs and more than three Aps were used and verified that least square lateration using many APs improves the accuracy of location estimations. However, if the estimated distance error between the AP and the device is large, it is important to select an AP that is capable of accurate distance estimation, because the additional APs may increase the localization error.

Zimu et al. [21] presented LiFi, which can be used to identify a LOS signal in a WiFi environment. LiFi can distinguish between LOS and NLOS with an accuracy rate of greater than 90% using the statistical characteristics of Orthogonal Frequency Division Multiplexing (OFDM) subcarriers. The channel state information (CSI) in the physical layer enables this analysis. This can be achieved without hardware modification, as described by the author. Wu et al. [22] proposed PhaseU, a real-time NLOS identification scheme for both static and mobile environments. PhaseU also exploits CSI, providing much finer-grained channel information than traditional RSSIs. By means of CSI, it observes the phase information to identify LOS and NLOS conditions. PhaseU achieves a LOS detection rate of 94.35%. Both LiFi and PhaseU require predefined thresholds that are variable in different environments; hence, they are difficult to use in practice. Li et al. [23] presented machine learning-based NLOS identification and mitigation for indoor WiFi localization. The proposed machine learning algorithm identifies NLOS by extracting several features of CSI. Even though the CSI-based NLOS identifications show very high accuracy, they are only applicable to limited WiFi network interface cards (NICs) providing CSI. No smartphone currently provides CSI to the user space; hence, these methods are not applicable in practice. Kim et al. [7] proposed a localization system that can identify LOS and NLOS using the attenuation characteristics of the 2.4 GHz and 5 GHz frequency bands. In an LOS environment, attenuation is constant, regardless of the frequency band. If the signal passes through obstacles, attenuation over NLOS paths depends on the frequency band. This simple method inspired our LOS and NLOS identification method.

## 5. Performance Evaluation

We present an evaluation of the performance of the proposed indoor localization algorithm in this section. The proposed localization algorithm was implemented as an Android application. We performed the experiment using an LG G2 smartphone and 3 ipTIME A604 WiFi APs on the fourth floor of building E10, Chungbuk National University.

The path loss threshold and path loss exponent, which are important parameters for the proposed indoor localization algorithm, were determined from the experiment. To determine the path loss threshold and path loss exponent, we first measured RSSIs at a distance of 5 m over each type of propagation path. The NLOS paths contained one concrete wall that was about 25 cm thick (NLOS level 1) or two concrete walls that were each about 25 cm thick (NLOS level 2). The RSSIs measured in the 2.4 GHz and 5 GHz bands were substituted into Equation (6) to obtain the path loss exponents in each band. Table 1 shows the measured RSSIs and the calculated path loss over the LOS and NLOS paths. Δ*RSSI* is the RSSI difference between the two bands obtained from the experiment. Δ*PL* was derived by substituting the calculated path loss exponent for each band into Equation (12). Δ*RSSI* and Δ*PL* were nearly identical. For this reason, we were able to estimate an accurate distance by means of LOS and NLOS identification.

**Figure 5:**
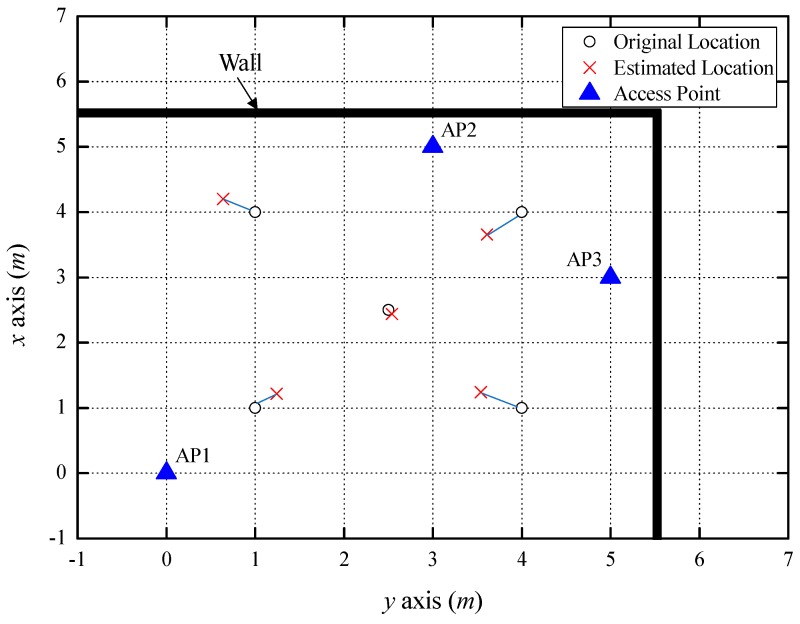
Indoor localization result using three LOS access points (APs.)

Figure 5 indicates the indoor localization results from the experiment using three LOS APs. For this experiment, we installed three APs and estimated the location of the smartphone in a room. The smartphone measured the RSSIs of the beacon frames received by each AP in the 2.4 GHz and 5 GHz bands. The RSSI difference between the 2.4 GHz and 5 GHz bands (Δ*RSSI*) was compared with the path loss threshold from Equation (13). Because the Δ*RSSI* for all APs did not exceed *PL_Thr1_*, the smartphone regarded the propagation path as LOS and estimated *d* from Equation (6). The average value of the estimated distances in the 2.4 GHz and 5 GHz bands was taken as the distance between the AP and the smartphone. The estimated distance errors at AP1, AP2, and AP3 were 0.27 m, 0.2 m, and 0.18 m, respectively. As a result of trilateration using the estimated distances, the average localization error was found to be 0.36 m at five coordinates. The error dropped to 0.07 m at the middle of the three APs (2.5, 2.5). Table 2 indicates the positions and errors estimated from the five coordinates.

**Figure 6:**
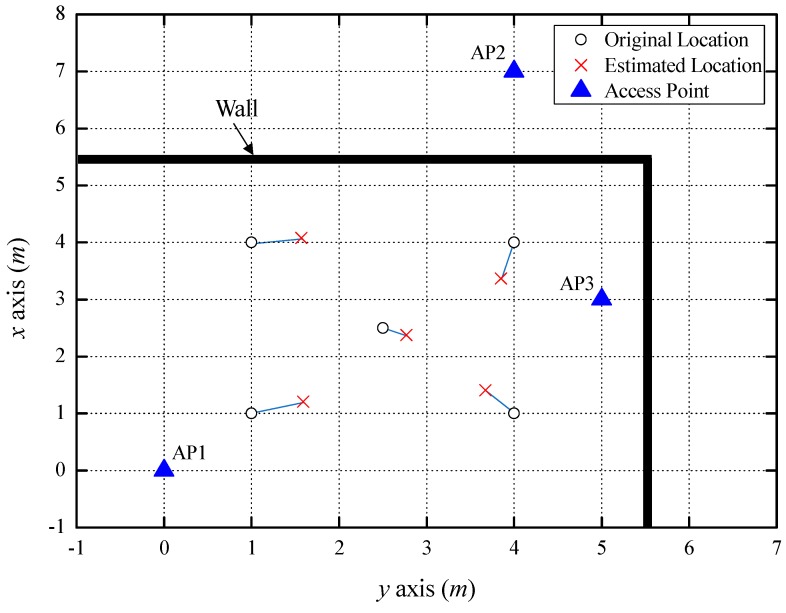
Indoor localization result using two LOS APs and one NLOS AP.

Figure 6 shows the indoor localization result using two LOS APs and one NLOS AP, separated by one concrete wall (NLOS level 1). The average estimated distance error between the smartphone and the APs was 0.36 m, which was increased by NLOS AP3. However, it was found that AP3 was NLOS level 1 from Equation (13), and the estimated distance error was reduced by using the path loss exponent, which is suitable for an NLOS level 1 environment. If the distance was estimated by applying a path loss exponent without distinguishing between LOS and NLOS, the estimated distance error would be larger. Although the estimated localization error became larger due to the increased estimated distance error, the accuracy was still within 1 m, on average. Table 3 shows the positions and errors estimated at five coordinates.

**Figure 7:**
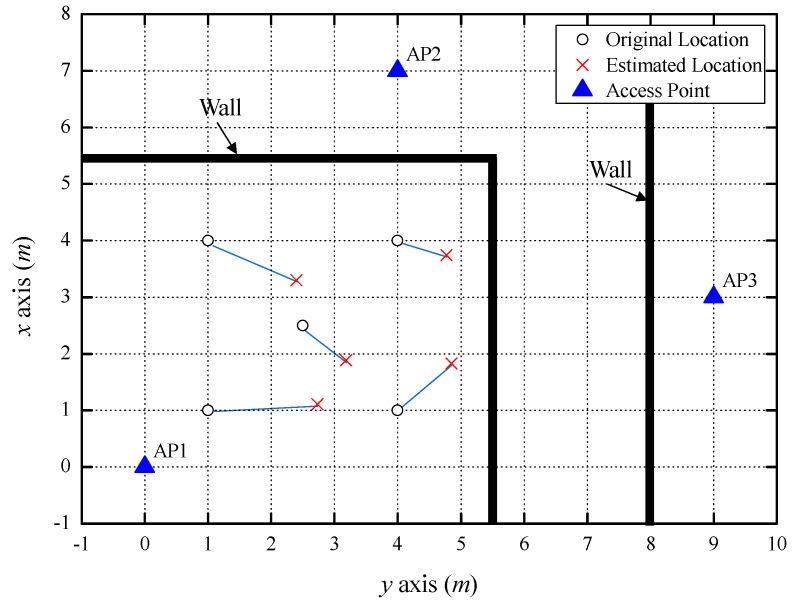
Indoor localization result using one LOS AP and two NLOS APs.

Figure 7 shows the indoor localization results when one LOS AP, one NLOS AP separated by one concrete wall (NLOS level 1), and one NLOS AP separated by two concrete walls (NLOS level 2) were used. In the NLOS environment, a larger number of obstacles resulted in larger RSSI variation due to shadowing. This made it difficult to predict the correct path loss exponent, resulting in a larger estimated distance error. For this reason, the average estimated distance error with three APs was 0.75 m, which was a considerable increase due to the estimated distance error for the NLOS level 2 path. The estimated distance was shorter than the actual distance between AP2 and the smartphone; thus, the estimated position shifted to the right, as shown in Figure 7; the average localization error was 1.24 m. Table 4 shows the positions and errors estimated from five coordinates.

**Figure 8:**
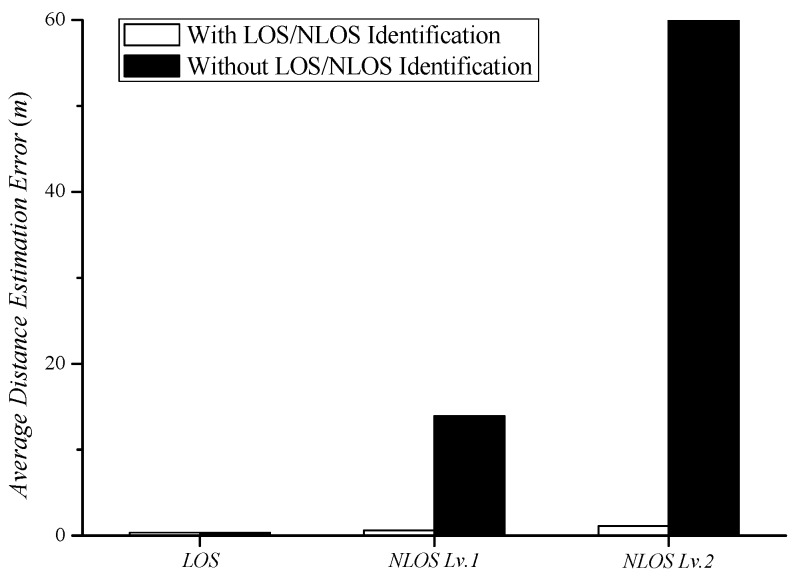
Average distance estimation error for different propagation paths.

Figure 8 shows the average distance estimation error along different propagation paths. Figure 5, Figure 6 and Figure 7 show the average distance estimation error along each propagation path. Traditional distance estimation methods do not consider whether the propagation path is LOS or NLOS; hence, they only use an LOS path loss exponent. If the real propagation path is LOS, then distance can be estimated with a low level of error. In the case of NLOS, the traditional distance estimation generated a large error causing it to exceed the experimental space (10 m × 10 m). Because the estimated distance is derived by Equation (6), it can go beyond the experimental space. The NLOS path has a large difference between the LOS path loss exponent and the real path loss exponent. The estimated distance is proportional to $10^{-n}$; thus, the use of the wrong path loss exponent, *n*, can cause a large error. The error caused by the NLOS level 2 AP is much larger than the error caused by the NLOS level 1 AP because the path loss exponent difference is large in the NLOS level 2 AP. In traditional distance estimation, the distance estimation error becomes larger as the number of NLOS APs or the difference in the path loss exponents increases. The proposed distance estimation method identifies LOS and NLOS using Equation (13). It finds and uses a proper path loss exponent according to the estimated propagation path. As in Figure 8, the proposed distance estimation method yields errors within 1.25 m, regardless of the propagation paths considered in trilateration.

**Figure 9:**
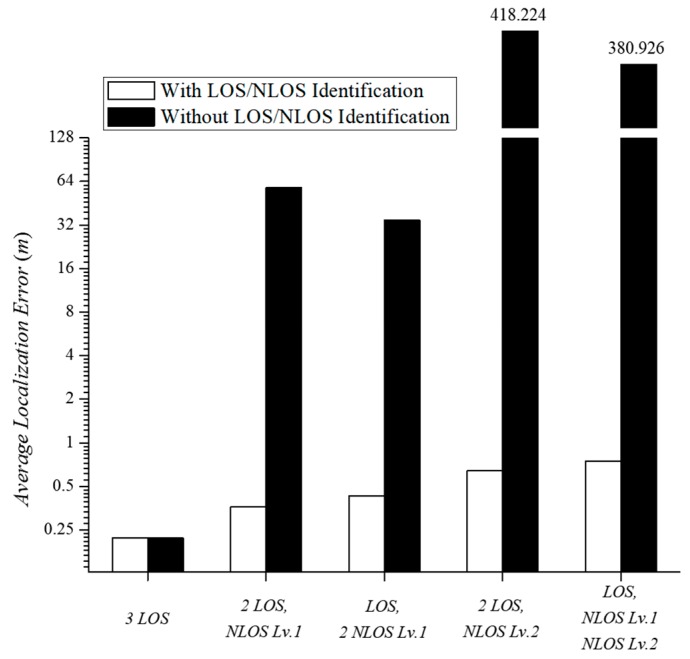
Average localization error in five experimental scenarios.

We investigated the results of trilateration based on the estimated distances between five different scenarios. As shown in Figure 9, the three LOS APs provided very accurate localization within an error of 0.36 m, regardless of LOS and NLOS identification. When the NLOS APs participated in indoor localization, the localization error increased due to the large distance estimation error incurred by NLOS APs. Using the traditional distance estimation, one NLOS level 1 AP generated an error of 13.92 m, resulting in a localization error of 58 m during trilateration. On the other hand, the use of two NLOS level 1 APs showed a rather low localization error. The error from each AP was canceled due to incorrect distance estimation from using each AP. The NLOS level 2 AP generated a considerably large localization error due to the large distance estimation error. That is, the use of trilateration without predicting the path loss exponent leads to a meaningless result beyond the experimental space (>10 m error). The results verify that trilateration-based localization with NLOS APs is impossible by means of the traditional distance estimation method. The proposed distance estimation method generated a low distance estimation error with an average error of 1.24 m or less, regardless of the propagation path. The proposed indoor localization algorithm is applicable to all RSSI-based localization technologies. This is expected to enhance the practicality of RSSI-based localization and enable actual LBS applications.

## 6. Conclusions

RSSI-based indoor localization was extensively studied for LBS applications. In RSSI-based indoor localization, it is important to accurately estimate the distance using RSSIs. In order to estimate the precise distance, a distance estimation model considering the shadowing effect is required. Many conventional studies have proposed solutions for accurate distance estimation, but most of them are difficult to implement in smartphone applications. In this paper, we proposed an accurate distance estimation model that accounts for shadowing. The proposed indoor localization algorithm diagnoses the propagation path as either LOS or NLOS and determines a proper path loss exponent for use in distance estimation. This allowed to the distances between the smartphone and the APs to be estimated more accurately. Based on the relatively accurate estimation of distance, we determined the location of a smartphone more precisely with trilateration localization. The experimental results showed that it is possible to reduce the localization error to within 1 m when more than two LOS APs exist and to within 1.5 m when one LOS AP exists around the smartphone. The proposed algorithm can be implemented as a smartphone application without hardware or software platform modification. This practical localization algorithm is expected to be applied in many smartphone-based LBS applications. In the future, we will verify whether the proposed algorithm is applicable to various experiments and applications.

## Figures and Tables

**Table 1:** Measured received signal strength indicator (RSSI) differences and estimated path loss over LOS and non-line-of-sight (NLOS) paths.

	RSSI		Δ*RSSI*	Path Loss Exponent		Δ*PL*
	2.4 GHz	5 GHz		2.4 GHz	5 GHz	
LOS	−41.21	−44.38	3.17	2.003	2.146	3.00
NLOS Lv. 1	−48.53	−61.77	13.24	3.796	4.831	13.61
NLOS Lv. 2	−55.88	−74.34	18.46	4.847	6.63	18.84

**Table 2:** Indoor localization result with three LOS APs.

Original Location	(1, 1)	(1, 4)	(2.5, 2.5)	(4, 4)	(4, 1)
Estimated Location	(1.24, 1.22)	(0.64, 4.2)	(2.54, 2.44)	(3.61, 3.66)	(3.54, 1.24)
Localization Error (*m*)	0.33	0.41	0.07	0.52	0.48
Average Total Error (*m*)	0.36				

**Table 3:** Indoor localization result with two LOS APs and one NLOS AP.

Original Location	(1, 1)	(1, 4)	(2.5, 2.5)	(4, 4)	(4, 1)
Estimated Location	(1.24, 1.22)	(0.64, 4.2)	(2.54, 2.44)	(3.61, 3.66)	(3.54, 1.24)
Localization Error (*m*)	0.33	0.41	0.07	0.52	0.48
Average Total Error (*m*)	0.36				

**Table 4:** Indoor localization result with one LOS AP and two NLOS APs.

Original Location	(1, 1)	(1, 4)	(2.5, 2.5)	(4, 4)	(4, 1)
Estimated Location	(2.73, 1.11)	(2.4, 3.3)	(3.18, 1.89)	(4.77, 3.74)	(4.85, 1.83)
Localization Error (*m*)	1.73	1.57	0.91	0.81	1.19
Average Total Error (*m*)	1.24				
